# Hypoglycemia associated with median arcuate ligament syndrome surgery

**DOI:** 10.1210/jcemcr/luag215

**Published:** 2026-08-10

**Authors:** Anvay Raje, Helen M Lawler

**Affiliations:** University of Colorado School of Medicine, Aurora, CO 80045, USA; Division of Endocrinology, Diabetes, and Metabolism, University of Colorado School of Medicine, Aurora, CO 80045, USA

**Keywords:** median arcuate ligament syndrome (MALS), hypoglycemia, celiac ganglionectomy, glucose metabolism, hyperinsulinemia

## Abstract

We present a patient who developed symptomatic hypoglycemia following surgical treatment for median arcuate ligament syndrome (MALS). The patient experienced predominately postprandial hypoglycemia after higher carbohydrate meals. Clinical findings revealed postprandial hyperinsulinemic hypoglycemia possibly related to celiac ganglionectomy performed during MALS surgery and non-insulin-mediated fasting hypoglycemia likely because of poor glycogen stores from restricting carbohydrates. Management consisted of dietary counseling, continuous glucose monitoring, and acarbose administered with meals. Following intervention, continuous glucose monitoring demonstrated improvement in hypoglycemia and overall improved glycemic stability. To our knowledge, this represents a novel report of hypoglycemia occurring after MALS surgery, highlighting a previously underrecognized metabolic consequence of celiac ganglionectomy.

## Introduction

The etiology of hypoglycemia in the absence of diabetes mellitus is heterogeneous and often difficult to diagnose. Associated autonomic manifestations—including tremors, diaphoresis, and palpitations—typically serve as early warning signs of hypoglycemia. Recurrent hypoglycemic episodes can cause neuroglycopenic symptoms, reflecting inadequate cerebral glucose availability, which may manifest as behavioral changes, impaired cognition, seizures, loss of consciousness, and, in severe cases, coma [1]. Given the substantial morbidity associated with these episodes, a comprehensive understanding of the pathophysiology underlying hypoglycemia is critical for accurate diagnosis and management.

**Figure 1:**
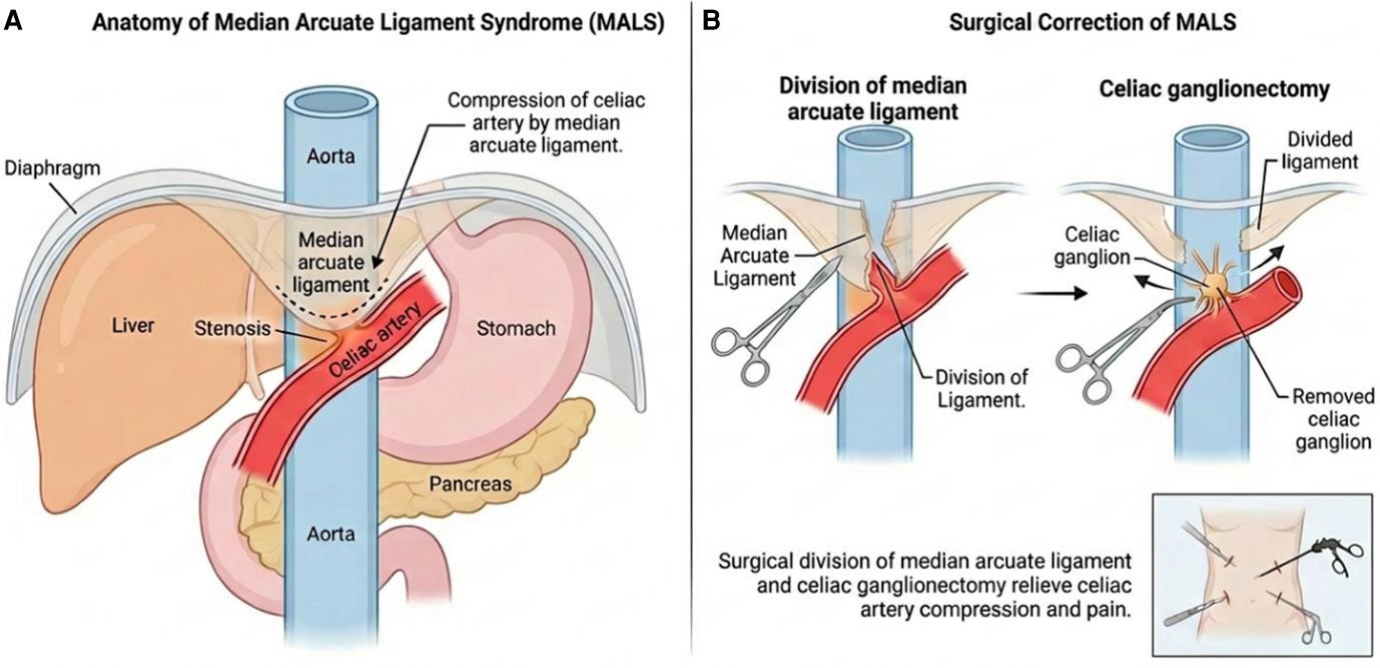
(A, B) Median arcuate ligament syndrome (MALS): anatomy and surgical correction (created with FigureLabs) [4]. (A) Median arcuate ligament and anatomical structures; (B) surgical steps of MALS surgery and celiac ganglionectomy to relieve artery compression.

Hypoglycemia without diabetes may be broadly classified into insulin-mediated and non-insulin-mediated etiologies. Insulin-mediated causes involve autonomous insulin secretion, most notably from insulinoma and other forms of endogenous hyperinsulinism such as noninsulinoma pancreatogenous hypoglycemia syndrome (NIPHS), postbariatric hypoglycemia, and insulin autoimmune syndrome. In contrast, non-insulin-mediated hypoglycemia may arise from conditions such as critical illness, organ failure, adrenal insufficiency, malnutrition, and non-islet cell tumors [2, 3]. We report a patient with postprandial hypoglycemia occurring after surgery to correct median arcuate ligament syndrome (MALS), a condition in which the median arcuate ligament compresses the celiac artery and celiac plexus (Fig. 1A). This compression leads to abdominal pain, nausea, vomiting, and postprandial pain, especially during exhaling [5]. MALS treatment involves surgical release of the ligament, removal of the abdominal nerve plexus, and potential vascular reconstruction to repair any stenosis or ischemia caused by the compression (Fig. 1B) [6].

## Case presentation

A 25-year-old female without previous history of hypoglycemia and medical history including hypermobile Ehlers-Danlos syndrome, postural orthostatic tachycardia syndrome, menorrhagia, and MALS underwent successful median arcuate ligament release with celiac ganglionectomy. The patient's surgery and hospital course were unremarkable. Postoperatively, she was able to tolerate a broader range of foods, although her weight remained stable, with no change in body mass index (BMI; 22 kg/m^2^) before or after surgery. Approximately 1 month after MALS surgery, she experienced a symptomatic hypoglycemic episode with vomiting after eating peanut butter crackers and mashed potatoes. Her capillary glucose was 42 mg/dL (2.3 mmol/L, reference range: 70-100 mg/dL; 3.9-5.6 mmol/L) and serum glucose was 43 mg/dL (2.4 mmol/L) in the emergency department.

**Figure 2:**
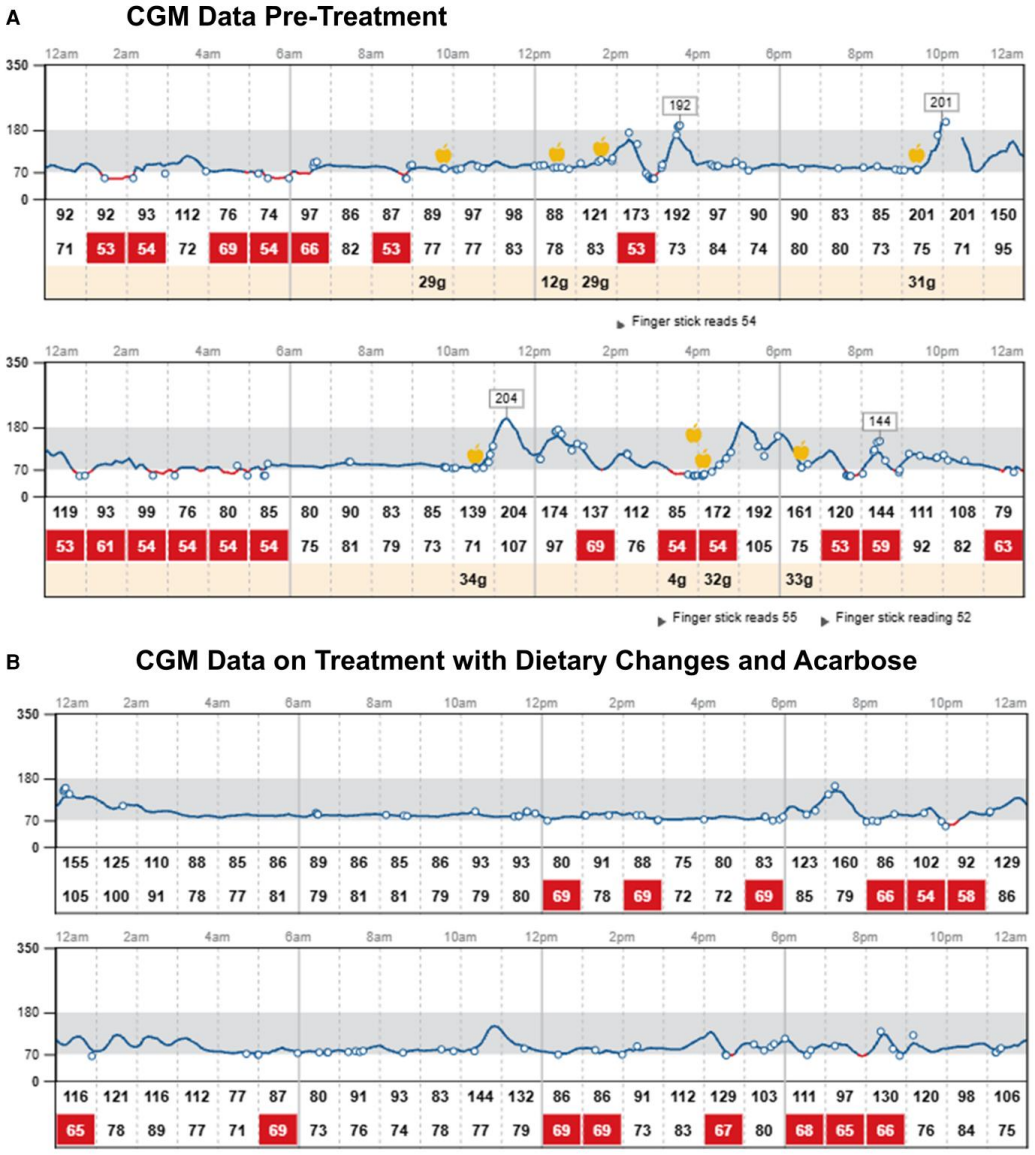
(A) Continuous glucose monitor (CGM) data pretreatment; (B) CGM data on treatment with a high-protein, low-carbohydrate diet, and acarbose 25 to 50 mg with meals.

The patient continued to experience intermittent hypoglycemia, particularly after consuming meals high in simple carbohydrates, with symptoms of tremulousness, diaphoresis, blurred vision, and difficulty concentrating. Continuous glucose monitor (CGM) data, which were confirmed via capillary glucose testing, showed postprandial hyperglycemia, often >200 mg/dL (11.1 mmol/L), followed by subsequent hypoglycemia with glucose values <55 mg/dL (<3.1 mmol/L) (Fig. 2A).

The patient's family history did not include hypoglycemia or MALS but did include maternal obesity and type 2 diabetes mellitus and a sibling with Ehlers-Danlos syndrome.

## Diagnostic assessment

In the clinic, vital signs were normal with a blood pressure of 123/82, pulse 95, temperature 36.9 °C (98.5 °F), respiration 16, oxygen saturation 100%, weight 54 kg (119 lb), height 1.575 m (5 ft 2 in), BMI 22 kg/m^2^. Physical examination revealed hypermobility with no other significant findings. Laboratory testing revealed human insulin antibody within reference range (<0.4 U/mL; reference range: 0-0.4 U/mL); insulinoma-associated protein 2 antibody within reference range (<5.4 U/mL; reference range: 0-7.4 U/mL); hemoglobin A1c within reference range (5.2%; reference range: 4.0-5.6%); and high 8 Am cortisol on oral contraceptive pill (28.2 µg/dL [778.0 nmol/L]; reference range: 4.5-22.7 µg/dL; 138.0-634.6 nmol/L). She declined discontinuation of her oral contraceptive pill to permit repeat cortisol testing and endorsed a good appetite, stable weight, normal bowel movements, and an absence of orthostatic symptoms, chronic nausea, or vomiting.

A 72-hour fast was also conducted to rule out insulinoma and drug-induced hypoglycemia. This revealed non-insulin-mediated hypoglycemia with a serum glucose of 54 mg/dL (3.0 mmol/L) at 24 hours, likely from carbohydrate restriction causing poor glycogen stores (Table 1). The 1-mg intravenous glucagon stimulation test showed a blunted glucose response (<25 mg/dL [<1.4 mmol/L] rise), suggesting depleted hepatic glycogen. She was symptomatic with adrenergic symptoms during hypoglycemia and experienced dizziness and vomiting after rescue with dextrose.

**Table 1:** Results of the supervised inpatient 72-hour fast and mixed meal tolerance test

Parameter	Patient results 24 hours into 72-hour inpatient fast	Reference range
Serum blood glucose	54 mg/dL (3.0 mmol/L)	70-99 mg/dL (3.9-5.6 mmol/L)
β-hydroxybutyrate (BHB)	45.08 mg/dL (4.33 mmol/L)	<2.81 mg/dL (<0.27 mmol/L)
Insulin	2 µIU/mL (12 pmol/L)	2-23 µIU/mL (36-180 pmol/L)
Proinsulin	0.29 µIU/mL (<2.0 pmol/L)	<1.04-1.15 µIU/mL (<7.2-8.0 pmol/L)
C-peptide	0.4 ng/mL (0.13 nmol/L)	0.9-5.2 ng/mL (0.30-1.72 nmol/L)
Sulfonylurea screen	None detected	None detected
Glucagon stimulation test	54 mg/dL (3 mmol/L) to 63 mg/dL (3.5 mmol/L) at 30 minutes	
Parameter	Patient results during mixed meal tolerance test	Reference range
Baseline glucose	87 mg/dL (4.8 mmol/L)	65-110 mg/dL (3.9-5.6 mmol/L)
Maximum glucose	166 mg/dL (9.2 mmol/L)	65-110 mg/dL (3.9-5.6 mmol/L)
Maximum insulin	169 µIU/mL (1014 pmol/L)	2-23 µIU/mL (36-180 pmol/L)
Lowest glucose	79 mg/dL (4.4 mmol/L)	65-110 mg/dL (3.9-5.6 mmol/L)

In addition, a mixed-meal tolerance test (MMTT) was conducted, where the patient consumed an Ensure Plus nutritional drink with glucose, insulin, c-peptide, proinsulin, and β-hydroxybutyrate tested over the course of 5 hours. MMTT results demonstrated, when compared to baseline, a robust insulin response of 169 µIU/mL (1174.55 pmol/L, reference range 2-23 µIU/mL; 36-180 pmol/L) and a significant glucose excursion of 87 mg/dL (4.8 mmol/L), although the blood glucose remained above 55 mg/dL (3.1 mmol/L) (Table 1). Last, a gastric emptying study was obtained and it was normal.

Overall, the patient's diagnostic results were consistent with postprandial hyperinsulinemic hypoglycemia and fasting non-insulin-mediated hypoglycemia.

## Treatment

The patient received dietary counseling to maintain a high-protein, low-carbohydrate diet (≤30 g carbohydrates per meal) while eliminating simple sugars. She continued CGM and was started on acarbose 25 to 50 mg 3 to 4 times daily with meals. Following treatment, CGM data demonstrated improvement in postprandial glycemic excursions as well as a reduction in fasting hypoglycemia, consistent with improved overall glycemic stability (Fig. 2B). A glucagon-like peptide-1 (GLP-1) receptor agonist was also considered as potential therapy to reduce glycemic variability; however, given the patient's normal BMI and concerns regarding treatment-associated weight loss, this option was not pursued.

## Outcome and follow up

The patient continues to have stable outpatient clinical status, has returned to work, safely operates a motor vehicle, and has not had any subsequent emergency department visits. She continues to experience intermittent postprandial hypoglycemia, primarily with carbohydrate intake exceeding 30 g per meal or consumption of simple sugars.

## Discussion

**Figure 3:**
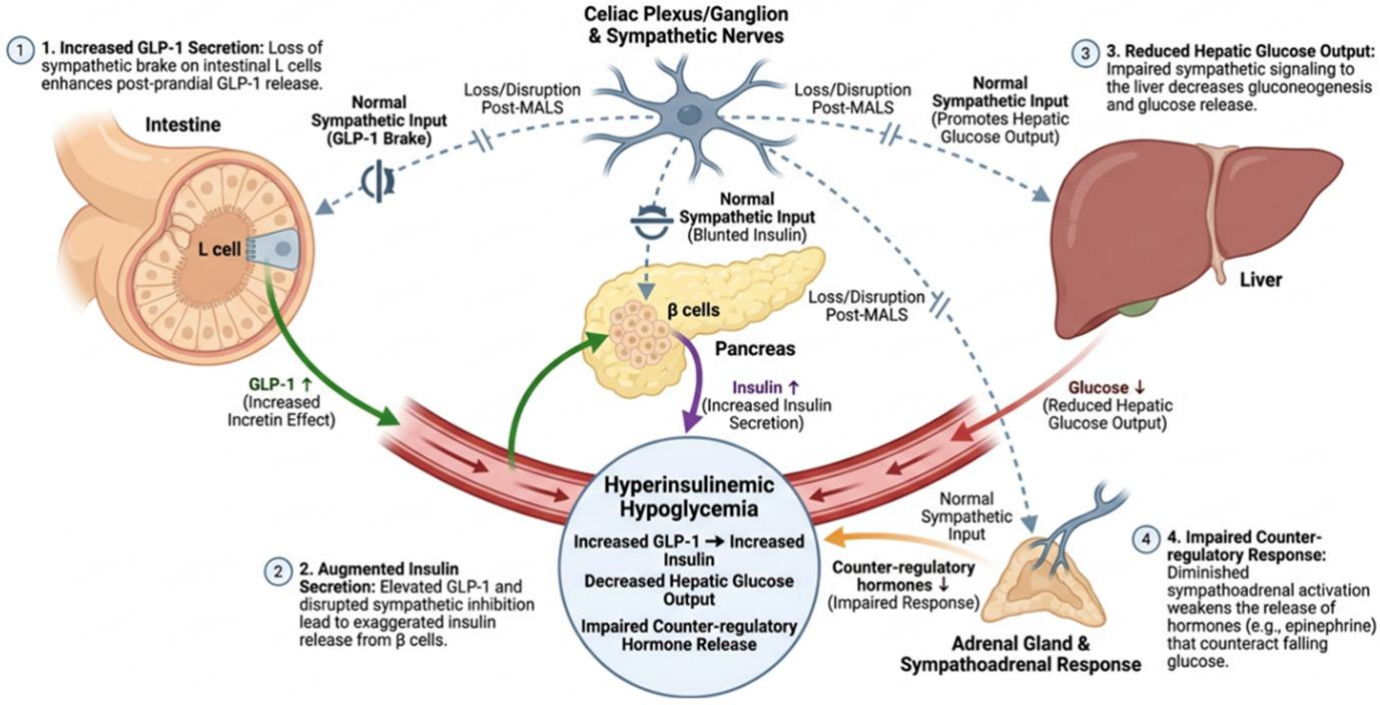
Multihit model of hypoglycemia in celiac ganglionectomy from MALS surgery (created with FigureLabs) [4]. Diagram illustrating a potential mechanism of post-MALS surgery hyperinsulinemic hypoglycemia, showing interactions between the intestine, pancreas, adrenal gland, and liver. Key elements include increased GLP-1 secretion from L cells, augmented insulin secretion by pancreatic β cells, reduced hepatic glucose output, and impaired counterregulatory hormone release.

The onset of hypoglycemia occurred shortly after MALS surgery and was not attributable to any established or conventional etiologies of hypoglycemia. Although multiple mechanisms may contribute to the development of hypoglycemia following surgery for MALS, celiac ganglionectomy appears to be a significant contributing factor (Fig. 3). Removal of the celiac ganglion could lead to a loss of sympathetic inhibition of insulin release, as evidenced by an in vitro human model showing celiac nerve stimulation resulted in inhibited sympathetic insulin release primarily via α-adrenergic mechanisms [7]. In MALS patients, the removal of the celiac ganglion could attenuate insulin release inhibition, possibly contributing to hyperinsulinemia and subsequent hypoglycemia. This potentially could be a mechanism for the exaggerated insulin secretion during this patient's MMTT.

Beyond the disinhibition of insulin release, celiac ganglionectomy could also enhance GLP-1 secretion postprandially, increasing the hyperinsulinemic response to nutrients. The celiac ganglion provides sympathetic innervation primarily via α2-adrenergic activation to enteroendocrine L cells, where it exerts inhibitory regulation of GLP-1 secretion [8]. Celiac denervation augments GLP-1 release from intestinal L cells, leading to hyperinsulinemia and hypoglycemia; sympathetic activation of the same nerve bundle showed opposite results [9]. This mechanism is particularly relevant in our patient's case, given the carbohydrate-triggered nature of her hypoglycemia.

Another consequence of MALS surgery is the altered hepatic handling of glucose. Removal of the celiac ganglion in an animal model reduced gluconeogenesis and lowered postprandial oral glucose tolerance test glucose peaks [9, 10]. The primary consequence of impaired gluconeogenesis is impaired mobilization of glucose from glycogen stores, resulting in decreased endogenous glucose production and less counterregulatory glucose supply, increasing susceptibility to hypoglycemia.

Compounding the impaired counterregulatory response to hypoglycemia, removal of the celiac ganglion in animal studies suppressed sympathetic adrenal response to hypoglycemia by up to 50%, whereas a vagotomy had no effect [11]. As a result, Fujita et al concluded that hypoglycemic detection at the portal vein is provided by glucose-sensing spinal pathway afferents via the celiac ganglion, not the vagal nerve. Thus, it is possible that ganglionectomy impaired the sympathetic counterregulatory response to hypoglycemia in our patient.

NIPHS is another potential mechanism for this patient's hypoglycemia. NIPHS patients present similarly with postprandial hyperinsulinemic hypoglycemia from nesidioblastosis, an abnormal proliferation, and enlargement of pancreatic β cells [12]. NIPHS was considered in this case given the possibility MALS surgery could have caused autonomic changes unmasking NIPHS pathophysiology. However, because the patient's hypoglycemia improved with dietary intervention and acarbose therapy, a selective calcium stimulation test was not performed to assess for possible nesidioblastosis.

Finally, rapid nutrient delivery following celiac ganglionectomy may cause exaggerated postprandial glucose excursions, excessive insulin secretion, and subsequent hypoglycemia a few hours after eating [3, 13, 14]. This is consistent with findings that 44% of patients with autonomic dysfunction have rapid gastric emptying, suggesting possible sympathetic effects of the celiac ganglion on gastric motility [15]. Thus, rapid emptying was considered, yet, our patient had a normal gastric emptying study.

Given the interdependence of glucose metabolism on sympathetic innervation by the celiac plexus, the recurrent hypoglycemia in this patient is likely related to the celiac ganglionectomy performed during MALS surgery. To our knowledge, this represents the first reported case describing postprandial hyperinsulinemic hypoglycemia and non-insulin-mediated fasting hypoglycemia following MALS surgery. Further investigation is warranted to determine the prevalence of hypoglycemia among patients with MALS, elucidate the underlying pathophysiological mechanisms, and develop targeted interventions to mitigate its clinical impact.

## Learning points

- MALS surgery can cause postprandial hypoglycemia
- Removal of the celiac ganglion during MALS surgery may contribute to hypoglycemia via changes in sympathetic mediation of glucose metabolism
- Management of hypoglycemia in this patient population includes dietary modification, CGM monitoring, and acarbose

## Data Availability

Original data generated and analyzed during this study are included in this published article.
